# The Efficacy and Safety of Use of Cannabis and Cannabinoid Products for Pain Relief in Orthopaedic Conditions and Trauma

**DOI:** 10.7759/cureus.87208

**Published:** 2025-07-03

**Authors:** Dhruvil Shah, Siddhartha Murhekar, Michael Ward, Shrikant Sagane, Ashvin Pahwani

**Affiliations:** 1 Trauma and Orthopaedics, King's College Hospital, London, GBR; 2 Trauma and Orthopaedics, Medway NHS Foundation Trust, Gillingham, GBR; 3 Orthopaedics, King's College Hospital, London, GBR; 4 Trauma and Orthopaedics, Medway Maritime Hospital, Gillingham, GBR; 5 Medicine, Jinnah Sindh Medical University, Karachi, PAK

**Keywords:** alternative pain therapies, analgesia, cannabis sativa, medical marijuana, opioid alternative

## Abstract

This systematic review examines the efficacy of medical cannabis in pain management within orthopaedic domains, including arthritis pain, postsurgical pain, back pain, and post-trauma pain. Given the challenges and risks associated with traditional pain medications, particularly opioids, this review aims to assess the efficacy and safety of medical cannabis for orthopaedic pain management.

A literature search was conducted on databases such as PubMed and Cochrane to find primary research papers on the efficacy and safety of cannabis. A comprehensive analysis was conducted on available literature, focusing on studies that evaluated the efficacy and safety profile of medical cannabis in various orthopaedic pain conditions. Only randomised controlled trials (RCTs) were included to keep the evidence of high quality. The quality of the studies was assessed with the Grading of Recommendations Assessment, Development, and Evaluation (GRADE) tool, and the risk of bias was assessed using the Cochrane Risk of Bias tool. The review particularly assessed the effectiveness of medical cannabis compared to no treatment, placebo, and active comparators. Additionally, the review examined the optimal dosing, methods of administration, and the safety profile of medical cannabis.

The review reveals minimal high-quality evidence supporting the efficacy of medical cannabis in the targeted orthopaedic areas. Most evidence suggests effectiveness only when compared with no treatment or placebo, with limited data against active comparators. The review also highlights the need for more research to determine optimal dosing and administration methods. The safety profile of medical cannabis, characterised by generally mild to moderate adverse effects, suggests its potential as a safer alternative or adjunct to opioid pain management.

The findings indicate that while medical cannabis may hold promise as an alternative or adjunct therapy in orthopaedic pain management, there is a need for more robust and methodologically sound research. Future studies should focus on long-term efficacy and safety, standardisation of dosing and administration, and comprehensive reporting of adverse effects. This is essential for developing effective treatment protocols that balance pain relief with safety and understanding the role of medical cannabis in orthopaedic pain management.

## Introduction and background

Pain is the most common symptom associated with different orthopaedic conditions and one of the leading causes of patients seeking medical attention [1]. Pain associated with orthopaedic conditions poses a significant global health burden, affecting millions worldwide [1]. This burden is complex, involving physical disability, psychological distress, and substantial healthcare costs. Musculoskeletal disorders, including those necessitating orthopaedic intervention, are identified as the second highest contributor to global disability, highlighting the extensive impact of this issue [1]. Additionally, lower back pain, a prevalent orthopaedic complaint, stands as the foremost cause of disability globally, cutting across various age groups and socio-economic sectors [2]. The prevalence and impact of orthopaedic pain are further intensified by aging populations and the rising incidence of chronic conditions. This underscores an urgent need for effective management strategies [3]. The financial burden of pain in orthopaedic conditions is substantial, straining healthcare systems and patients alike [4]. A 2010 study by Clay et al. titled "Bio-psychosocial determinants of time lost from work following non-life threatening acute orthopaedic trauma" examines the variables that determine how long an employee will miss work after an acute orthopaedic non-life-threatening trauma. This study, which involved 168 patients and four hospitals in Victoria, Australia, emphasises the significant effect orthopaedic injuries have on missed work and the resulting financial costs [5]. The management of pain in orthopaedic patients presents a complex challenge due to its potential to be both chronic and acute, encompassing nociceptive, inflammatory, and neuropathic types. With the global trend of ageing populations, there is a growing apprehension about the escalating burden of pain associated with orthopaedic ailments [6].

"Cannabis" refers to a range of drugs derived from the *Cannabis *genus of plants [7]. This term encompasses various substances used for both medicinal and recreational purposes. Cannabis ranks among the most widely used recreational drugs globally [8]. In 2012, it was estimated that approximately 178 million individuals aged 15-64 years consumed cannabis at least once [8]. The United Nations' Single Convention on Narcotic Drugs in 1961 classified cannabis as a controlled substance. Consequently, its usage remains illegal in the majority of countries worldwide [9].

The second and third steps of the World Health Organisation's step ladder for pain management involve the use of opioids [10]. Compared to opioids, cannabinoids have emerged as a possible safer alternative for the treatment of pain [11]. The search for safer analgesics has become necessary due to the opioid crisis, which is characterised by widespread addiction and overdose mortality [12]. The opioid crisis in the United States (US) and the United Kingdom (UK) represents a significant public health challenge. In the US, the crisis is particularly severe, with around 47,600 opioid-related deaths reported in 2017, as per the Centers for Disease Control and Prevention (CDC) [13]. This epidemic largely stems from the overprescription of opioids in the late 1990s, fuelled by assurances from pharmaceutical companies about their non- addictive nature [12]. In the UK, the situation mirrors this troubling trend, albeit on a smaller scale. Based on results from England and Wales' 2014-15 Crime Survey, 5.4% of adults between the ages of 16 and 59 reported abusing a prescription painkiller that was not written for them [14]. The Office for National Statistics recorded 2,208 opioid-related deaths in England and Wales in 2018, the highest since records began [15]. Factors contributing to this include overprescription and inadequate monitoring [15].

According to Nielsen et al. (2017), cannabinoids, which are derived from the cannabis plant, interact with the body's endocannabinoid system to provide analgesic effects without the substantial danger of addiction and overdose that comes with opioids [16]. In addition, compared to opioids, cannabinoids have been shown to have fewer and milder adverse effects [16]. They are therefore a good choice for people who want pain relief without running the danger of abusing opioids. The capacity of cannabis to reduce a variety of pain states, such as neuropathic and inflammatory pain, provides evidence of its medicinal promise in pain treatment [17]. The legalisation of medical and recreational cannabis in Canada and some US states coincides with the growing need for safe pain management strategies in orthopaedic conditions [18]. Osteoarthritis, affecting 27 million in the US, and back pain, impacting a quarter of Americans, exemplify the widespread nature of orthopaedic pain [19]. With an ageing global population, the burden of orthopaedic pain is expected to rise [20]. The therapeutic applications of cannabinoids have been explored in various clinical trials, focusing on their potential to alleviate symptoms in several medical conditions [21]. These include managing insomnia [22], nausea and vomiting associated with cancer chemotherapy [23], addressing appetite loss in individuals with weight loss due to human immunodeficiency virus (HIV) or cancer [24], treating chronic pain [25], reducing spasticity in multiple sclerosis (MS) patients [25], controlling intraocular pressure in glaucoma cases [26], and providing relief in other conditions like spinal cord injury (SCI) [27].

The International Association for the Study of Pain (IASP) established a task force in 2018 to explore cannabis and cannabinoid analgesia [28]. Recent research shows a surge in medical cannabis studies, with over half of the 9057 citations in the US National Library of Medicine from the past five years [29]. Cannabinoids have Food and Drug Administration (FDA) approval for a specific condition and are being investigated for various applications, including chronic pain and orthopaedic surgery [29].

Madden et al.'s 2018 systematic review highlighted the use of cannabinoids in orthopaedic surgery, noting their potential in post-operative recovery and opioid reduction [30]. With widespread opioid misuse in the USA and UK [14,31], cannabinoids offer a promising alternative. Their widespread use is demonstrated by the fact that 14% of Americans used cannabidiol (CBD) products in 2019 for pain relief, and 31.2% of adults in England and Wales between the ages of 16 and 59 reported using cannabis at least once in their lifetime [32-33]. The meta-analysis and systematic review conducted by Whiting et al. revealed moderate-quality evidence in favour of cannabinoids being used to treat chronic pain. In multiple trials, they saw a significant decrease in pain compared to placebo [34]. In the context of orthopaedic conditions, preliminary evidence shows promising results. A study conducted by Blake et al. and colleagues indicated that rheumatoid arthritis patients experienced significant pain relief when using cannabis [35-37].

In terms of existing systematic reviews, the review by Whiting et al. 2015 concluded that evidence of moderate quality supports the effectiveness of cannabinoids in treating chronic pain and spasticity. Meanwhile, evidence of lower quality indicates potential benefits of cannabinoids in alleviating chemotherapy-induced nausea and vomiting, promoting weight gain in HIV patients, improving sleep disorders, and helping with Tourette syndrome [34]. A review by Madden et al. focused on study methodology for trials regarding pain in orthopaedics [30]. Vivance et al. concluded in their review of therapeutic studies that to further establish the analgesic effects, rigorous evidence from well-designed randomised controlled trials tailored to orthopaedic surgery was required [29]. Another review by First et al. suggested that more focused clinical research was needed to determine the effectiveness of cannabinoid products as an analgesic for low-back pain and associated symptoms, despite the substantial body of research on their use for many medical conditions, including the treatment of chronic pain [38]. Price et al.'s review on low back pain suggested that even though the effectiveness of cannabis in treating low back pain with a reasonable side effect profile was established, there was a need for high-quality studies with longer follow-ups [39]. A review on analgesia in rheumatic conditions by Fitzcharles et al. concluded that while there were positive outcomes on pain relief and sleep with mild to moderate side effects, the research could benefit from studies of larger sample sizes, longer follow-up, and lesser heterogeneity in the data [40].

The present literature concludes the positive effects of analgesia in orthopaedic conditions with acceptable side effects, but suggests studies of higher quality of evidence. The necessity for this systematic review arises from several gaps in the existing research on medical cannabis use, particularly in orthopaedic conditions. While there is a substantial amount of evidence available, previous studies have fallen short in determining the most effective type, dosage, and administration method of cannabis [30]. Moreover, earlier systematic reviews have not specifically concentrated on orthopaedic conditions. A recent scoping review focusing on the use of medical cannabis for musculoskeletal pain highlighted the requirement for more high-quality research in this area. Additionally, existing studies in this field often exhibit unclear or high risks of bias, and the evidence is generally rated as low or very low quality according to the Grading of Recommendations Assessment, Development, and Evaluation (GRADE) system. The objective of this study was to summarise the literature on the efficacy and safety of cannabis use for analgesia in orthopaedic conditions. The focus was mainly on the inclusion of randomised controlled trials (RCTs), which can contribute to higher-quality evidence.

While previous reviews have explored the role of cannabinoids in managing chronic pain broadly, they often amalgamate data from diverse conditions such as fibromyalgia, cancer pain, and multiple sclerosis. These conditions may differ significantly in pathophysiology and response to cannabinoids compared to orthopaedic and trauma-related pain. Consequently, the conclusions drawn from such heterogeneous populations may not be directly applicable to orthopaedic patients. There remains a noticeable lack of systematic reviews that focus specifically on randomised controlled trials evaluating cannabis-based interventions in clearly defined orthopaedic or trauma-related pain conditions. This review aims to address that gap by synthesising evidence strictly from RCTs involving orthopaedic and trauma-related pain, providing more targeted insights for clinical practice.

## Review

Methodology

**Figure 1:**
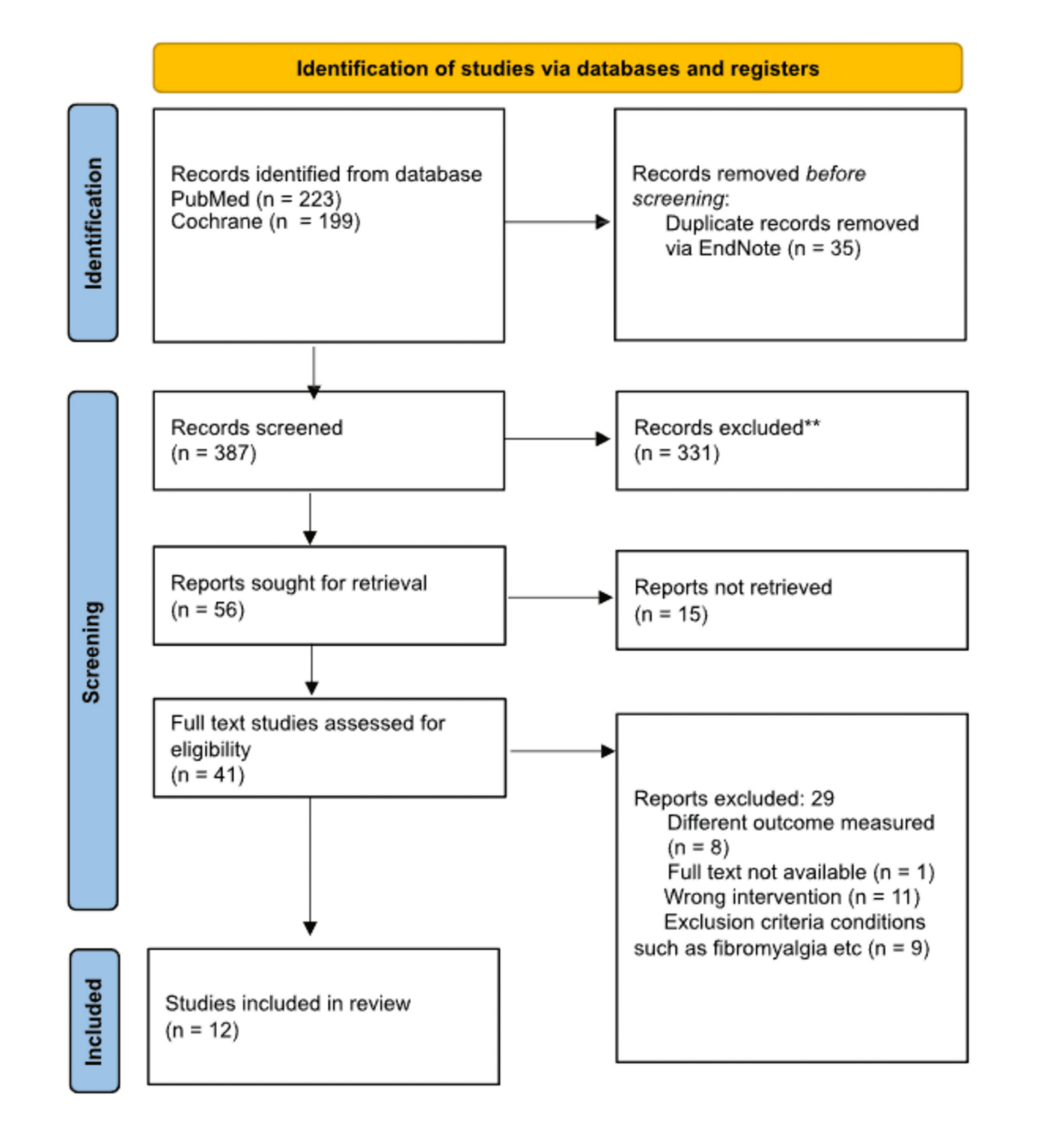
Flowchart diagram of study identification and selection process ** excluded after screening through the titles

Figure 1 below highlights the study identification and the selection process.

Objectiv

The primary objective of this systematic review was to analyse high-quality primary evidence concerning the efficacy and safety of cannabis and cannabinoid products in alleviating pain for various orthopaedic conditions and neurological pain caused due to trauma.

Search Strategy and Selection Criteria

A comprehensive search strategy was conducted using the PubMed and Cochrane databases to identify studies on cannabinoids for orthopaedic and trauma-related pain. The PubMed search used the following search string: ((((((orthopaedics) OR (orthopedics)) OR (trauma)) OR (injury)) OR (pain)) OR (injury)) AND (((((cannabis) OR (cannabinoids)) OR (thc)) OR (sativex)) OR (nabilone)). This search yielded 223 results from PubMed. For the Cochrane database, the search strategy used was: "orthopaedics OR orthopedics OR trauma OR injury OR pain OR injury AND cannabis OR cannabinoids OR thc OR sativex OR nabilone AND efficacy and safety", which produced 199 results. In total, 422 records were identified. After removing 35 duplicate records using EndNote software, 387 unique records remained for screening. Following title and abstract screening, 331 records were excluded, leaving 56 reports sought for full-text retrieval. Of these, 15 reports could not be retrieved. A total of 41 full-text reports were assessed for eligibility. Among these, 29 reports were excluded for the following reasons: different outcomes measured (n=8), full text not available (n=1), wrong intervention (n=11), exclusion criteria conditions such as fibromyalgia and other unrelated pain conditions (n=9). Ultimately, 12 studies met the inclusion criteria and were included in this systematic review.

Inclusion criteria were limited to randomised double-blinded controlled trials published within the past 20 years (2003-2023), focusing on orthopaedic conditions causing pain, such as acute and chronic back pain, rheumatoid arthritis, and neuropathic pain resulting from trauma or injury. Some papers with neuropathic pain in the title were included after full-text research revealed the cause of pain as trauma or injury. Primary efficacy criteria were centred on pain scores. Exclusion criteria encompassed studies related to sensory neuropathy, multiple sclerosis, diabetic neuropathy, fibromyalgia, post-surgical pain, cancer pain, and other non-cancer pain causes, as well as studies involving cannabinoid mimetic interventions such as palmitoylethanolamide and fatty acid amide hydrolase (FAAH) inhibitors. Also, papers in languages other than English were also excluded.

Study Selection Process

From the initial pool of 422 papers, titles and abstracts were screened based on the inclusion and exclusion criteria, followed by a full-text review for selected studies.

Data Extraction and Analysis

Data extraction was performed manually from the primary studies. Extracted data points included study characteristics (such as design, duration, and setting), total population size, patient demographics, types of cannabinoids used, modes of administration, primary efficacy criteria (specifically pain scores and percentage improvement from baseline), and primary safety criteria (including adverse event incidence and the most common mild to moderate adverse effects). Secondary outcomes such as effects on mood, cognition, and sleep quality were also documented.

Data extraction and risk of bias assessments were performed independently by two reviewers, with a third resolving disagreements.

The methodology employed ensured a rigorous, systematic approach to identifying and synthesising relevant literature, with a focus on providing a comprehensive overview of the current state of evidence regarding the efficacy and safety of cannabis and cannabinoid products in orthopaedic pain management.

In the methodology section of the systematic review, rigorous standards were employed to evaluate the included studies. A critical component of the analysis was the use of the Cochrane Risk of Bias Tool. This tool was utilised to systematically assess the risk of bias in each study, focusing on various aspects such as random sequence generation, allocation concealment, blinding of participants and personnel, blinding of outcome assessment, incomplete outcome data, selective reporting, and other biases. Each study was meticulously evaluated against these criteria to ensure a comprehensive and unbiased review of the available evidence. Additionally, the Grading of Recommendations, Assessment, Development, and Evaluations (GRADE) approach was adopted to assess the overall quality of evidence from the studies. The GRADE framework allowed the evaluation of the quality of evidence based on factors like study design, consistency of results, directness of evidence, precision of estimates, and publication bias. By applying these robust methodologies, the systematic review was conducted with a high degree of scientific rigour, providing clear and reliable conclusions drawn from the evaluated literature.

Results

After conducting a thorough literature search, 422 potentially relevant articles were identified from electronic databases such as PubMed and Cochrane. Out of which 387 articles were considered for screening after removing 35 duplicates in EndNote. Forty-one full-text articles were assessed for eligibility. All 12 selected were double-blinded, randomised, controlled trials [37,41-51].

Methodologic Quality and Bias

The Cochrane Risk of Bias tool was used to assess the methodological quality of randomised trials. The Grading of Recommendations Assessment, Development and Evaluation (GRADE) guidelines were utilised to assess the credibility of selected RCTs (Table 1).

**Table 1:** GRADE assessment of the primary studies GRADE - Grading of Recommendations Assessment, Development and Evaluation

Sr No	Author	Types of studies included	Risk of bias	Inconsistency	Indirectness	Imprecision	Publication bias	Quality of evidence
1	Almog et al., 2020 [41]	Randomised, three-armed, double-blind, placebo-controlled, crossover study	Not serious	Not serious	Not serious	Serious	Not serious	Moderate
2	Bebee et al., 2021 [42]	Single-centre, randomised, double-blinded, placebo-controlled clinical trial	Not serious	Not serious	Not serious	Serious	Not serious	Moderate
3	Berman et al., 2004 [43]	single centre, double- blind, randomised, placebo-controlled, three-period crossover study	Not serious	Not serious	Not serious	Serious	Not serious	Moderate
4	Blake et al., 2006 [37]	Randomised, double- blind, parallel group study	Not serious	Not serious	Not serious	Serious	Not serious	Moderate
5	Frank et al., 2008 [44]	Double-blind randomised controlled study	Not serious	Not serious	Not serious	Serious	Not serious	Moderate
6	Karst et al. 2003 [45]	Randomised, placebo-controlled, double-blind crossover trial	Not serious	Not serious	Not serious	Serious	Not serious	Moderate
7	Nurmikko et al., 2007 [46]	Randomised, double-blind, placebo-controlled parallel group study	Not serious	Not serious	Not Serious	Serious	Not serious	Moderate
8	Rintala et al., 2010 [47]	Randomised, double-blind, crossover, controlled study	Not serious	Not serious	Not serious	Serious	Not serious	Moderate
9	Ware et al., 2010 [48]	Randomised, double- blind, placebo-controlled, four-period crossover design.	Not serious	Not serious	Not serious	Serious	Not serious	Moderate
10	Wilsey et al., 2008 [49]	Double-blinded, placebo-controlled, crossover study	Not serious	Not serious	Not serious	Serious	Not serious	Moderate
11	Wilsey et al., 2013 [50]	Randomised, double-blind, placebo-controlled, crossover design	Not serious	Not serious	Not serious	Serious	Not serious	Moderate
12	Wilsey et al., 2016 [51]	Randomised, placebo-controlled, crossover trial	Not serious	Not serious	Not serious	Serious	Not serious	Moderate

In the systematic review, the GRADE assessment of included randomised controlled trials (RCTs) revealed a diverse range of evidence quality across various studies. Overall quality of the study was graded as moderate.

While most studies were rated as high to begin with because of being RCTs, they were later downgraded because of imprecision for having a sample size <400 for continuous data (pain scores).

The risk of bias assessment of the included studies in this systematic review reveals a mixed level of quality. Several studies demonstrated low risk in key domains such as sequence generation, allocation concealment, and blinding of participants and assessors, indicating a high standard of methodological quality. Specifically, studies by Bebee et al. (2021), Nurmikko et al. (2007), Rintala et al. (2010), and Wilsey et al. (2008, 2013) consistently showed low risk across most categories [42,46,47,49,50,52].

Conversely, there were instances where critical information was not provided, as seen in studies by Almog et al. (2020), Berman et al. (2004), Blake et al. (2005), Karst et al. (2003), and Ware et al. (2010) [37,41,45,48]. The lack of information in essential areas such as sequence generation, allocation concealment, and blinding raises concerns about potential biases in these studies. Additionally, some studies exhibited a high risk of bias in specific areas. For instance, the study by Karst et al. (2003) showed a high risk of bias in incomplete outcomes, which could potentially impact the reliability of the results [45]. The study by Blake et al. (2006) was funded by GW Pharmaceuticals, and some authors received honoraria from GW Pharmaceuticals for their work on the study design and protocol development [37]. This financial connection could introduce a potential source of bias.

Table 2 below highlights the Cochrane Risk of Bias for all included RCTs.

**Table 2:** Cochrane Risk of Bias

Study	Sequence generation	Allocation concealment	Blinding participants	Blinding assessors	Incomplete outcomes	Selective reporting
Almog et al., 2020 [41]	No information	No information	Low	No information	Low	Low
Bebee et al., 2021 [42]	Low	Low	Low	Low	Low	Low
Berman et al., 2004 [43]	No information	Low	Low	No information	Low	No information
Blake et al., 2006 [37]	No information	No information	Low	Low	Low	Low
Frank et al., 2008 [44]	No information	No information	Low	no information	High	Low
Karst et al. 2003 [45]	No information	No information	Low	Low	High	No information
Nurmikko et al., 2007 [46]	Low	Low	Low	No information	No information	No information
Rintala et al., 2010 [47]	Low	Low	Low	No information	Low	Low
Ware et al., 2010 [48]	No information	No information	Low	No information	No information	No information
Wilsey et al., 2008 [49]	Low	Low	Low	Low	No information	No information
Wilsey et al., 2013 [50]	Low	Low	Low	No information	Low	No information
Wilsey et al., 2016 [51]	Low	No information	No information	No information	Low	No information

Study Characteristics 

The studies varied in their focus, ranging from chronic pain associated with rheumatic diseases (Almog et al. 2020) to specific conditions like avulsion of nerve rootlets (Berman et al. 2004) and neuropathic pain post-traumatic nerve lesion (Nurmikko et al. 2007) [41,43,46].

Study designs were predominantly randomised and double-blind, emphasising a strong methodological approach. However, the specifics of the designs varied, including crossover studies (Frank et al. 2008, Karst et al. 2003), parallel group studies (Blake et al., Nurmikko et al. 2007), and clinical trials (Bebee et al. 2021) [37,44-46].

Table 3 below highlights the study characteristics for the included studies.

**Table 3:** Study characteristics table CBD - cannabidiol; THC - tetrahydrocannabinol; CT3 - 1',1'dimethylheptyl-Delta8-tetrahydrocannabinol-11-oic acid; RCT - randomised controlled trial

Study	Patient characteristics	Study design	Total patients (N)	Type of cannabinoid
Almog et al., 2020 [41]	Chronic pain associated with rheumatic diseases, including back pain and osteoarthritis	Randomised, three-armed, double-blind, placebo-controlled, crossover study	27	THC aerosol
Bebee et al., 2021 [42]	Adults with acute, non-traumatic low back pain	Single-centre, randomised, double-blinded, placebo-controlled clinical trial	100	CBD
Berman et al., 2004 [43]	Avulsion of nerve rootlets from the spinal cord following traction injuries to the brachial plexus	Single-centre, double-blind, randomised, placebo-controlled, three-period crossover study	48	Sativex
Blake et al., 2006 [37]	Rheumatoid arthritis	Randomised, double-blind, parallel group study	58	Sativex
Frank et al., 2008 [44]	Neuropathic pain syndrome post-injury or surgery	Double-blind randomised controlled trial	48	Nabilone
Karst et al. 2003 [45]	Chronic neuropathic pain due to traumatic nerve lesion	Randomised, placebo-controlled, double-blind crossover trial	21	Synthetic cannabinoid CT-3
Nurmikko et al., 2007 [46]	Neuropathic pain post-traumatic nerve lesion	randomised, double-blind, placebo-controlled parallel group study	125	Sativex (2.7 mg THC and 2.5 mg CBD)
Rintala et al., 2010 [47]	Spinal cord injury	Randomised, double-blind, crossover, controlled study	7	Dronabinol 5 mg
Ware et al., 2010 [48]	Adults with post-traumatic or postsurgical neuropathic pain	Randomised, double-blind, placebo-controlled, four-period crossover design.	21	Tetrahydrocannabinol herbal cannabis
Wilsey et al., 2008 [49]	Complex regional pain syndrome (type I), spinal cord injury, peripheral neuropathy, or nerve injury	Double-blinded, placebo-controlled, crossover study	38	High-dose cannabis (7% delta-9-THC), low-dose cannabis (3.5% delta-9-THC)
Wilsey et al., 2013 [50]	Neuropathic pain	Randomised, double-blind, placebo-controlled, crossover design	39	Tetrahydrocannabinol herbal cannabis
Wilsey et al., 2016 [51]	Neuropathic pain from spinal cord injury	A randomised, placebo-controlled crossover trial	42	2.9%, and 6.7% THC

Patient Characteristics

The patient groups addressed different pain conditions, like chronic neuropathic pain (Karst et al. 2003), rheumatoid arthritis (Blake et al.), and spinal cord injuries (Rintala et al. 2010) [37,45,47]. The number of participants varied significantly across studies, from as few as 7 in Rintala et al. (2010) to as many as 125 in Nurmikko et al. (2007), indicating a range in the scale and potentially the power of these studies [46,47].

Mode of Administration

Inhalational methods were used in Almog et al. (2020) and Ware et al. (2010), while oral administration was chosen by Bebee et al. (2021), Frank et al. (2008), and Rintala et al. (2010) [41,48,42,44]. Oromucosal sprays were utilised in Berman et al. (2004), Blake et al., and Nurmikko et al. (2007) [37,43,46]. These different modes of administration reflect diverse approaches in cannabinoid delivery, potentially influencing efficacy and patient tolerance.

Study Period

The study duration ranged from a brief 150 minutes in Almog et al. (2020) to longer periods of four to five weeks in Berman et al. (2004), Blake et al., and Nurmikko et al. (2007). This variability in study periods could impact the assessment of both immediate and long-term effects of cannabinoids on pain [37,43,46].

Treatment and Dosage

The studies used a range of cannabinoid treatments, from aerosolised Δ9-THC (delta-9-tetrahydrocannabinol), CBD, and Sativex, to synthetic cannabinoids like CT-3 (1',1'-dimethylheptyl-Delta8-tetrahydrocannabinol-11-oic acid) and nabilone [37,41-43,46]. Doses varied, reflecting the tailored approach to different pain types and patient needs.

Control Group

Placebo was commonly used as a control across studies, providing a baseline for comparing the efficacy of the cannabinoid treatments. However, Frank et al. (2008) and Rintala et al. (2010) used active comparators instead of a placebo [44,47].

Efficacy Outcome Measured Scale

Pain scales like Visual Analogue Scale (VAS), Numeric Rating Scale (NRS), and box score 11 (BS-11) were used to quantify pain relief, allowing a standardised assessment of efficacy across different patient populations and pain conditions. The scales usually ranged from 0-10 or 0-100. Hence, efficacy outcomes of 30% and 50% reduction in pain intensity were reported for percentage improvement in pain scores from the baseline. The proportion of people with at least 30% pain intensity reduction/moderate improvement and the proportion of people with at least 50% pain intensity reduction/substantial improvement as defined by the Initiative on Methods, Measurement, and Pain Assessment in Clinical Trials (IMMPACT) [52,53].

Table 4 concludes the primary efficacy outcomes for the studies included.

**Table 4:** Primary efficacy outcome table with results (+) - treatment has superior analgesic control than comparator; (-) - treatment does not have superior analgesic control than comparator; (?) - inclusive outcome; CBD - cannabidiol; THC - tetrahydrocannabinol; CT3 - 1',1'dimethylheptyl-Delta8-tetrahydrocannabinol-11-oic acid; RCT - randomised controlled trial; VAS - Visual analogue Scale; NRS - Numerical Rating Scale GW-2000-02 / GW-1000-02 – study codes for formulations of Sativex (specific doses/formulations in Berman et al.)

Study	Mode of administration	Study period	Treatment and dosage	Control	Efficacy outcome measured scale	% change from baseline for treatment group	% change from baseline for control group	Result of efficacy outcome	Overall effect
Almog et al., 2020 [41]	Inhalational	150 minutes	A single inhalation of 0.5 mg and 1.0 mg aerosolised Δ9-THC	Placebo	VAS score (0 -10)	25% (0.5 mg) and 38.82% (1 mg)	Data for drop in placebo group not given	Both doses cause significant pain reduction compared to baseline. Higher dose (1 mg) caused significant pain reduction compared to placebo	+
Bebee et al., 2021 [42]	Oral	120 minutes	400mg CBD	Placebo	Verbal numerical pain scale (0-10)	16.67%	17.24%	CBD was not superior to placebo as an adjunct medication	-
Berman et al., 2004 [43]	Oromucosal spray	4 weeks	Sativex oromucosal spray containing 27 mg/ml THC and 25 mg/ml CBD	Placebo	BS-11 score (0-10)	GW-2000-02 = 16% GW- 1000-02 = 18.67%	Baseline data for placebo not given	p=0.02 for GW-2000-02 and p=0.005 for GW-1000-02, BS-11 pain severity score, did not decrease by the two points as specified in hypothesis. However, statistically significant improvements were seen in both this measure and sleep-related measures.	+
Blake et al. [37]	Oromucosal spray	5 weeks	Sativex (2.7 mg THC and 2.5 mg CBD)	Placebo	NRS (0-10)	31.43 % % in pain on movement, 41.51% in pain on rest	20.9% in pain on movement, 22.64% in pain on rest	A significant analgesic effect was observed and disease activity was significantly suppressed	+
Frank et al., 2008 [44]	Oral	Treatment period 1 (six weeks), washout period (two weeks), and treatment period 2 (six weeks); 3 months	nabilone	Dihydrocodeine	VAS score (0-100)	13.89%	15.83%	Dihydrocodeine provided better pain relief than the synthetic cannabinoid nabilone	-
Karst et al. 2003 [45]	Oral capsule	5 weeks	Synthetic cannabinoid CT-3	Placebo	VAS score (0-100) and VRS	CT-3–Placebo Sequence at 11 AM: VAS Reduction: 28.84% VRS Reduction: 18.89% CT-3–Placebo Sequence at 4 PM: VAS Reduction: 26.75% VRS Reduction: 23.76% Placebo–CT-3 Sequence at 11 AM: VAS Reduction: 18.40% VRS Reduction: 21.49% Placebo–CT-3 Sequence at 4 PM: VAS Reduction: 16.59% VRS Reduction: 20.13%		CT-3 was effective in reducing pain compared to placebo	+
Nurmikko et al., 2007 [46]	Oromucosal spray	5 weeks	Sativex (2.7 mg THC and 2.5 mg CBD)	Placebo	NRS (0-10)	22%	8%	The mean reduction in pain intensity scores was greater in patients receiving sativex than placebo Numerical Rating Scale (p = 0.004) and Neuropathic Pain Scale composite score (p = 0.007)	+
Rintala et al., 2010 [47]	Oral	A 12-day upward titration phase. 7-day stabilisation phase. A 28-day maintenance phase. A 9-day downward titration phase. A 7-day washout phase.	Dronabinol 5 mg	Diphenhydramine	Pain scale (0- 10)	2.50%	22.50%	dronabinol was no more effective than diphenhydramine for relieving chronic neuropathic pain below the level of injury.	-
Ware et al., 2010 [48]	Inhalation	Each medication trial consisted of a 63-day period (12 days for titration, 7 days for stabilization, 28 days for maintenance, 9 days for downward titration, and 7 days for washout).	25 mg of 9.4% tetrahydrocannabinol herbal cannabis	Placebo	NRS (0-10)	2.5 mg group experienced a 3.28% decrease in pain, the 6 mg group experienced a 1.64% decrease, and the 9.4 mg group experienced an 11.48% decrease.	0	smoked cannabis reduces pain	+
Wilsey et al., 2008 [49]	Ihalation	Mean 7.8 days	high-dose cannabis (7% delta-9-THC), low-dose cannabis (3.5% delta-9-THC) machine-rolled into cigarettes	Placebo	VAS score (0-100)	both the 3.5% and 7% treatments resulted in a 112.5% greater reduction in pain intensity per minute compared to the placebo.	Baseline data for placebo not given	cannabis, at both low and high doses, was more effective than placebo	+
Wilsey et al., 2013 [50]	Vaporized cannabis	7 days	medium dose (3.53% delta-9-THC), low- dose (1.29% delta-9- THC	Placebo	VAS score (0-100)	(1.29% THC )7.13% reduction in pain compared to the placebo, and the (3.53% THC) treatment shows an approximate 0.35% reduction in pain compared to the placebo.		the study supports the efficacy of cannabis in reducing neuropathic pain, with both low and medium doses being more effective than placebo	+
Wilsey et al., 2016 [51]	Inhalation	8 hours	6.7% and 2.9% delta- 9- tetrahydrocannabinol (THC) vaporized	Placebo	The Neuropathic Pain Scale (0-10)	The 6.7% concentration group showed a 45% decrease in pain from the baseline (from 5 to 2.75).	The placebo group showed a 10% decrease in pain from the baseline (from 5 to 4.5).	while there were some indications of cannabis affecting pain outcomes, the evidence was not strong enough to be conclusive without considering the possibility of false positives	?

Percentage Change from Baseline

The percentage of pain reduction from baseline varied, with Almog et al. (2020) reporting up to 38.82% reduction, while Bebee et al. (2021) observed a 16.67% decrease with CBD [41,42]. This variation highlights the differential effectiveness of cannabinoids based on type and dosage. The systematic review's results, focusing on the percentage change in pain from baseline across different studies, reveal varied efficacy of cannabinoids in pain management. In Almog et al. (2020), aerosolised Δ9-THC demonstrated a significant dose-dependent reduction in pain, with 25% for 0.5 mg and 38.82% for 1 mg. Contrastingly, Bebee et al. (2021) showed that 400mg of oral CBD reduced pain by 16.67%, closely mirroring the placebo's 17.24%, indicating limited superiority over placebo. Studies employing Sativex, like Berman et al. (2004) and Blake et al., reported notable pain reductions of 16% to 18.67% and 31.43% to 41.51%, respectively, underscoring its effectiveness [37,43]. However, Frank et al. (2008) observed a slightly more significant pain reduction with dihydrocodeine (15.83%) compared to nabilone (13.89%). Karst et al. (2003), using synthetic cannabinoid CT-3, demonstrated varied pain reduction between 16.59% to 28.84%, suggesting its efficacy. Nurmikko et al. (2007) also found Sativex effective, with a 22% decrease in pain compared to 8% in the placebo group [44-46]. In contrast, Rintala et al. (2010) reported minimal efficacy with oral Dronabinol, showing only a 2.5% pain reduction compared to 22.5% in the control group. Ware et al. (2010), using different THC concentrations in herbal cannabis, noted an 11.48% pain reduction at the highest concentration (9.4% THC), suggesting a dose-response relationship [48]. Lastly, Wilsey et al. (2016) highlighted the significant efficacy of cannabis in reducing neuropathic pain, with one study showing a 112.5% greater reduction in pain intensity [51]. These findings collectively illustrate the diverse effectiveness of cannabinoids in pain management, varying across different types, dosages, and administration modes, with some showing substantial improvement over placebo, while others were less effective or comparable to placebo or active comparators.

Result of Efficacy and Safety Outcome

The results varied from significant pain reduction in Almog et al. (2020) and Blake et al., to less pronounced effects or no superiority over placebo as in Bebee et al. (2021) and Frank et al. (2008) [37,41,42,44].

Table 5 highlights the safety outcomes.

**Table 5:** Primary safety outcome T - treatment, C - control; AEE - adverse effect events; CBD - cannabidiol; THC - tetrahydrocannabinol; CT3 - 1',1'dimethylheptyl-Delta8-tetrahydrocannabinol-11-oic acid; RCT - randomised controlled trial GW-2000-02 / GW-1000-02 – Study codes for formulations of Sativex (specific doses/formulations in Berman et al.) ** serious side effect due to patient condition

Study	Serious side effects for treatment group	Side effects leading to withdrawal	Total AEE (T/C)	Conclusion	Dizziness (T/C)
Almog et al., 2020 [41]	1**	1	22 (0.5 mg) /14 AND 20 (1 mg) / 14	The majority of adverse events were minor and concluded on their own. No evidence of persistent deficits in cognitive function was found.	1 mg : 8 vs 1 0.5 mg : 2 vs 1
Bebee et al., 2021 [42]	1**	Data not given	(POST 48 hours) 20/26	Side effects were similar for the two groups	1 vs 1
Berman et al., 2004 [43]	0	3	GW-2000-02 31/13 and GW-1000-02 24/13	The study medication was well tolerated by all patients with no serious AEs	GW-2000-02 11 vs 4 GW-1000-02 9 vs 4
Blake et al. 2006 [37]	0 vs 2 (T/C)	0 vs 3 (T/C)	31/ 27	The large majority of adverse effects were mild or moderate, and there were no adverse effect-related withdrawals or serious adverse effects in the active treatment group	8 vs 1
Frank et al., 2008 [44]	Data not given	8 vs 4 (T/C)	(334/305)	Dihydrocodeine had slightly fewer side effects	Not given
Karst et al. 2003 [45]	Not mentioned	Not mentioned	CT-3–placebo sequence -6/0 placebo–CT-3 sequence 6/5	Did not find clinically relevant adverse effects	Not given
Nurmikko et al., 2007 [46]	2	11 vs 2	91% / 77%	The benefits of better sleep, decreased disability, and subjective pain relief outweighed the drawbacks of adverse events	18 vs 9
Rintala et al., 2010 [47]	1	1 vs 1 (T/C)	30 /18	Mentions various side effects experienced by participants in both groups	4 vs 3
Ware et al., 2010 [48]	0	1	(2.5 % - 61/46) (6 % - 65/ 46) (9.4 % - 82/ 46)	Well tolerated compared to placebo	(2.5% - 3/2) (6% - 4/2) (9.4% - 4/2)
Wilsey et al., 2013 [49]	0	0	Not mentioned	Psychoactive effects were minimal and well-tolerated	Not mentioned
Wilsey et al., 2008 [50]	0	0	Not mentioned	Psychoactive effects were minimal and well-tolerated	Not mentioned
Wilsey et al., 2016 [51]	0	0	Not mentioned	Does not explicitly state a conclusion about the safety outcome of the treatment	Not mentioned

Superiority Over the Control

The overall effect of cannabinoid treatments on pain management was generally positive, with most studies showing significant pain relief. Out of 12 RCTs, eight reported superiority over the control group (mainly placebo), three were non-superior, and one was inconclusive.

Discussion

The necessity for a systematic evaluation of cannabinoids effectiveness in treating pain in orthopaedic conditions stems from an increasing interest in alternative methods for pain management, especially given the ongoing opioid crisis conventional approaches to pain relief, which largely depend on opioids, have resulted in significant problems such as addiction, tolerance, and negative side effects, underscoring the need to investigate other, more reliable and safer options. It is believed that approximately 7.1 million adults in England are prescribed opioid or gabapentinoid pain medications [54]. Commonly prescribed drugs like codeine, morphine, and tramadol, often administered as immediate solutions following injuries or surgeries, carry risks of addiction and, in cases of misuse or extended use, can potentially lead to fatal outcomes [54]. Cannabinoids, known for their distinct mode of action compared to opioids, have been proposed as a viable treatment alternative. Current research, however, offers a scattered and inconsistent view of their effectiveness and safety. The demonstrated efficacy of cannabis-based medicines in reducing nausea and vomiting in chemotherapy patients, as shown by Smith et. al (2015), underscores their potential therapeutic value [55]. Given their comparable effectiveness to conventional antiemetics, exploring cannabis for pain relief becomes a logical next step. This is particularly relevant considering the ongoing search for safer, effective alternatives to traditional pain management methods, like opioids [56]. The success in one symptom control area suggests promising potential for cannabis in pain management [57].

There is a pressing need for a new systematic review in the realm of pain management, particularly addressing the efficacy and safety of different cannabinoids in treating pain related to trauma and orthopaedic conditions. Existing reviews have incorporated a wide array of study designs, including observational studies, non-controlled studies, and cross-sectional studies. These reviews have covered various pain conditions, ranging from neuropathic pain, cancer pain and fibromyalgia to chronic back pain and pain resulting from rheumatic diseases. However, there remains a significant gap in high-quality, systematic reviews that specifically focus on trauma and orthopaedic-related pain. Such a review would be invaluable, as it would provide more targeted and reliable information on the potential role of cannabinoids in these specific areas, helping to guide clinical decisions and inform future research directions in pain management within orthopaedic and trauma care settings.

Although this review only contains published RCTs, there remains a lot of heterogeneity among the study methods, interventions, mode of administration, primary efficacy outcome scale measurement, and analysis of safety profile. Cannabinoids, particularly those administered via oromucosal spray and inhalation, generally showed a trend of effective pain reduction. For instance, Blake et al. and Nurmikko et al. reported significant pain reduction with Sativex, an oromucosal spray. In contrast, oral administrations, such as in the studies by Bebee et al. and Frank et al., showed less pronounced effects, indicating that the mode of administration may play a critical role in efficacy.

A review by Aviram et al., which incorporated the results of 43 randomised controlled trials (RCTs) involving 2437 patients, found limited evidence showing more pain reduction in chronic pain, especially by inhalation [58]. This analysis indicated a significant reduction in pain scores with the inhalation method compared to placebo. However, the review also noted that while some RCTs showed clinically significant improvements, the majority did not demonstrate a noticeable effect, leading to uncertainty about the clinical significance of these findings [58]. Post-approval randomised trials of THC:CBD oromucosal spray, used for treating resistant multiple sclerosis spasticity, have confirmed its efficacy and safety [59]. These studies also addressed specific concerns of health authorities, showing no adverse effects on cognition and mood after prolonged treatment. The add-on therapy with THC:CBD spray was significantly more effective than adjusting standard antispasticity therapy alone [59]. These findings illustrate a complex picture where the mode of cannabinoid administration plays a crucial role in its efficacy for pain management. While inhalation methods have shown promise in certain studies, the use of oromucosal sprays like nabiximols has been backed by more extensive research and post-approval trials, suggesting a more consistent and reliable effect on chronic pain conditions, especially neuropathic pain. The disparity in the effectiveness of these methods underscores the need for further research to optimise cannabinoid-based treatments for pain management.

Higher THC concentrations tended to yield better pain relief. For example, Wilsey et al. (2008) using high-dose cannabis (7% THC) showed a significant reduction in pain, contrasting with lower THC 16 concentrations in other studies that reported lesser pain reduction [49]. Almong et al. reported higher pain reduction with a 1 mg dose compared to a 0.5 mg dose [41]. Ware et al. also reported the highest pain relief in the 9.4 mg group amongst the 2.5 and 6 mg groups [48]. This is supported by a systematic review categorising cannabinoids based on their THC-to-CBD ratio and source, assessing both synthetic products with high THC concentrations and extracted products. The findings revealed that synthetic products with a high THC-to-CBD ratio (>98% THC) might lead to moderate improvements in pain severity and response, albeit with an increased risk of sedation and a probable significant risk of dizziness [60]. Similarly, extracted products with high THC-to-CBD ratios (ranging from 3:1 to 47:1) may also be associated with an increased risk of adverse events and dizziness [60].

There is a noticeable difference in the percentage of pain reduction across studies. Almog et al. reported a substantial decrease in pain (up to 38.82%) with aerosolised Δ9-THC, while Bebee et al. observed only a marginal pain reduction with CBD, comparable to placebo [41,42]. A systematic review assessing cannabinoids categorised by THC-to-CBD ratio also found that synthetic products with high THC-to-CBD ratios (greater than 98% THC) are associated with moderate improvement in pain severity and response. In contrast, products with comparable THC-to-CBD ratios (1.1:1) showed smaller improvements in pain severity and overall function. This indicates that THC might be more effective than CBD in managing pain, especially considering the higher potency of THC in pain reduction observed in the studies.

Studies like Karst et al. and Rintala et al. showcased contrasting results despite both being oral administrations [45,47]. Karst et al.'s use of synthetic cannabinoid CT-3 showed a significant reduction in pain, whereas Rintala et al. observed minimal efficacy with Dronabinol [45]. This variation could be attributed to differences in the cannabinoid compounds used and the specific pain conditions treated.

The studies covered a range of pain conditions, from neuropathic pain due to spinal cord injury to chronic rheumatic pain. While cannabinoids showed efficacy in conditions like neuropathic pain (Wilsey et al. 2013, Nurmikko et al. 2007), the response in rheumatic pain (Blake et al.) and chronic back pain (Almog et al. 2020) was also notable [37,41,46]. This diversity suggests cannabinoids' potential as a versatile treatment option across various pain conditions. The efficacy of cannabinoids in treating specific chronic pain subtypes, including neuropathic pain, fibromyalgia pain, and geriatric pain, has been highlighted. However, a comprehensive review by Ang et al. demonstrated that they are less effective in acute postoperative pain and most musculoskeletal pain syndromes except fibromyalgia [11,61]. While cannabinoids have shown some positive effects in treating cancer pain, the results are not as conclusive [62]. Despite these findings, current evidence does not support cannabinoids as the first-line treatment for any type of acute or chronic pain [11].

Instead, they may be considered as an adjunct or alternative treatment for patients who have failed conventional measures. Cannabinoids have demonstrated potential as an alternative or adjunct treatment for chronic pain, offering a possibility for patients who have not found relief with conventional measures. Cannabinoids are emerging as a viable alternative or adjunct to opioids for pain management. A significant study with 2897 participants showed that 97% could reduce opioid use with cannabis, and 81% found it more effective alone for pain relief [63]. This highlights cannabis's potential in offering comparable pain relief to opioids but with fewer side effects [64]. Additionally, cannabinoids can enhance pain relief when used with opioids, reducing opioid dependence and potentially mitigating addiction-related issues [64]. This points towards the benefits of medical cannabis in managing chronic pain and addressing the challenges associated with pharmaceutical opioid use.

Most studies reported minimal to non-serious side effects in the treatment group, suggesting a generally favourable safety profile for cannabinoids.

While withdrawal due to side effects was almost negligible, it varied across studies. In some cases (e.g., Ware et al., Wilsey et al., Blake et al.), there were no adverse effect-related withdrawals in the treatment group, while other studies (e.g., Frank et al., Nurmikko et al.) reported higher withdrawal rates, indicating variability in tolerability [37,44,46,48,49].

There was a noticeable variation in total adverse effect events (AEE) between treatment and control groups across studies. For example, Almog et al. reported a higher AEE in the treatment group compared to the control, while Bebee et al. observed a similar incidence of side effects in both groups [41,42]. The common conclusion across these studies is that the adverse effects were mostly mild or moderate, and serious adverse events were rare or non-existent, which is consistent with the findings and 17 conclusion of other literature in this field of study [65].

Central nervous system adverse effects, such as sedation, euphoria, fatigue, mental clouding, disorientation, dizziness, somnolence, confusion, dissociation, and psychomotor deficits, are the most common symptoms associated with medical cannabis use. Lower doses of medical cannabis may help in reducing adverse effects while still preserving its analgesic properties, as these effects are dependent on the dosage [41]. Additionally, even at these reduced doses, neurocognitive side effects, including challenges with learning, memory, and psychomotor functions, may occur. However, these are typically mild, well-tolerated, and tend to resolve on their own [29,41].

Dizziness was a common adverse event in many studies; hence, it was used as a comparator among all 12 RCTs in this review. In several studies, the incidence of adverse events was higher in the treatment group than in the control group, which is expected given the pharmacological effects of cannabinoids. For instance, in the study by Blake et al., dizziness was more prevalent in the treatment group compared to the control [37]. This suggests that dizziness is a notable side effect associated with cannabinoid use.

Although not as extensively researched, the use of smoked cannabis has been linked to adverse outcomes like lung cancer [66] and reduced bone mineral density in heavy users [18]. In their study, Ware et al. focused on adverse effects (AEs) mainly related to smoked cannabis, observing a decline in lung function and a rise in upper respiratory issues and infections in their cannabis group over a year [67]. Smoking, a prevalent method of administration reviewed, raises several concerns due to the harmful effects of burning plant material, similar to the well-documented negative impacts of smoked tobacco [68,69].

Strengths and limitations

The systematic review in question exhibits several notable strengths, significantly enhanced by adherence to rigorous evaluation frameworks such as Grading of Recommendations, Assessment, Development, and Evaluations (GRADE) and the Cochrane Risk of Bias tool. The application of the GRADE system underscores the reliability of the findings, as it thoroughly assesses the quality of evidence, ensuring that the conclusions are grounded in robust data. This approach is particularly crucial in cannabinoid research, where the quality of evidence can vary substantially. By incorporating the Cochrane Risk of Bias tool, potential biases in the included studies are meticulously evaluated, enhancing the credibility of the review. This meticulous assessment aligns with the high standards recommended in the existing literature for systematic reviews in the medical field.

Furthermore, the diversity of studies included in the review, covering various cannabinoids, dosages, and administration routes, provides a comprehensive perspective on the potential and limitations of cannabinoid therapy. This broad scope aligns with calls in the literature for inclusive evaluations to better understand the nuances of cannabinoid therapies. The review's balanced focus on therapeutic effects and adverse events offers a well-rounded analysis, crucial in the evolving domain of cannabinoid research. The stringent inclusion and exclusion criteria in the methodology ensure that the analysed studies are relevant and of high quality, a practice highly valued in scientific literature.

The systematic review in question, while comprehensive, does encounter several limitations that warrant attention. Firstly, there is variability in the methodologies and quality of the studies included, impacting the consistency and comparability of the results. This challenge is commonly observed in cannabinoid research, as highlighted in other literature, where studies often vary significantly in dosage, route of administration, patient demographics, and the condition being treated. Furthermore, many studies feature small sample sizes or short durations, limiting the generalizability of their findings. This aspect is particularly relevant in cannabinoid research, where long-term effects and potential risks might not be fully understood through short-term studies. Another significant limitation is the potential bias in reporting adverse events and outcomes. In some instances, the subjective nature of self-reported data can introduce bias, and the illegal status of cannabis in many regions further complicates accurate data collection and reporting. Additionally, the lack of standardisation in the types and strains of cannabis used across studies leads to varied pharmacological profiles and consequently different therapeutic and adverse effects. This lack of standardisation represents a critical gap as identified in the broader literature, underscoring the need for more controlled and standardised research in this field.

Future recommendations

The future of cannabinoid research necessitates a comprehensive and multifaceted approach, 18 addressing the current gaps and enhancing our understanding of cannabinoid-based therapies. Key areas for future studies include conducting large-scale, long-term research to evaluate the efficacy and safety of cannabinoids, especially in chronic conditions, and implementing more standardised practices in terms of types, strains, dosages, and administration routes of cannabinoids. Enhanced study designs, such as randomised controlled trials with larger, diverse patient groups, are crucial for generating reliable data. Exploring the broader impacts of cannabinoids on physical, emotional, and cognitive health, and understanding their pharmacological mechanisms for pain relief, are also essential. Research should focus on cannabis as a potential alternative or adjunct to traditional pain medications like opiates, assessing its role in pain relief, side effect profiles, and the management of opiate withdrawal symptoms. Prioritising patient-centred outcomes, such as quality of life and functional improvement, will provide deeper insights into the benefits and drawbacks of cannabis use. Furthermore, addressing legal, social, and ethical considerations in different regions and ensuring comprehensive reporting of both acute and chronic adverse effects are vital. Incorporating objective measures to validate self-reported outcomes and conducting economic analyses to evaluate cost-effectiveness will also enrich the field. By addressing these diverse aspects, future research can significantly contribute to the evolving understanding of cannabinoids in medical therapy and pain management, potentially offering safer and more effective treatment strategies.

## Conclusions

The available high-quality evidence supporting the effectiveness and safety of medical cannabis and cannabinoids in managing pain due to trauma and key orthopaedic conditions is limited. Current studies predominantly indicate the efficacy of medical cannabis when compared to no treatment or a placebo, rather than to an active comparator. Consequently, the actual effectiveness of medical cannabis in these areas remains uncertain. It is also evident that cannabinoids present a relatively favourable safety profile, especially when compared to traditional pain medications like opioids. The existing literature, though limited in some aspects, consistently indicates that the adverse effects associated with medical cannabis are generally mild to moderate in nature. These effects commonly include neurologic symptoms such as dizziness and sedation, gastrointestinal disturbances, and mild cognitive impairments. However, the potential for use of cannabinoids as an adjunct should be further explored. The diverse methodologies used for research contribute to challenges in determining consistent form, administration routes, dosing and frequency. This variability hinders the development of standardised treatment protocols. To address these gaps, future studies should focus on enhancing the quality of reporting and research methods. This improvement will be crucial in developing treatment protocols that effectively manage pain while minimising potential adverse effects. Such advancements in research will provide clearer guidelines and a better understanding of the role of medical cannabis in orthopaedic pain management.
